# Evaluation of inflammation-related genes polymorphisms in Mexican with Alzheimer’s disease: a pilot study

**DOI:** 10.3389/fncel.2015.00148

**Published:** 2015-05-18

**Authors:** Danira Toral-Rios, Diana Franco-Bocanegra, Oscar Rosas-Carrasco, Francisco Mena-Barranco, Rosa Carvajal-García, Marco Antonio Meraz-Ríos, Victoria Campos-Peña

**Affiliations:** ^1^Departamento de Fisiología Biofísica y Neurociencias, Centro de Investigación y de Estudios AvanzadosMexico City, Mexico; ^2^Posgrado en Ciencias Biológicas, Universidad Nacional Autónoma de MéxicoMexico City, Mexico; ^3^Instituto Nacional de GeriatríaMexico City, Mexico; ^4^Hospital Regional de Alta Especialidad de IxtapalucaEstado de México, Mexico; ^5^Centro Geriátrico SINANK’AYQuerétaro, Mexico; ^6^Departamento de Biomedicina Molecular, Centro de Investigación y de Estudios AvanzadosMexico City, Mexico; ^7^Laboratorio Experimental de Enfermedades Neurodegenerativas, Instituto Nacional de Neurología y Neurocirugía Manuel Velasco SuárezMexico City, Mexico

**Keywords:** Alzheimer’s disease, inflammation, prostaglandin-endoperoxide synthase, cyclooxygenase 2, genetic ancestry

## Abstract

Amyloid peptide is able to promote the activation of microglia and astrocytes in Alzheimer’s disease (AD), and this stimulates the production of pro-inflammatory cytokines. Inflammation contributes to the process of neurodegeneration and therefore is a key factor in the development of AD. Some of the most important proteins involved in AD inflammation are: clusterin (CLU), complement receptor 1 (CR1), C reactive protein (CRP), tumor necrosis factor α (TNF-α), the interleukins 1α (IL-1α), 6 (IL-6), 10 (IL-10) and cyclooxygenase 2 (COX-2). In particular, COX-2 is encoded by the prostaglandin-endoperoxide synthase 2 gene (PTGS2). Since variations in the genes that encode these proteins may modify gene expression or function, it is important to investigate whether these variations may change the developing AD. The aim of this study was to determine whether the presence of polymorphisms in the genes encoding the aforementioned proteins is associated in Mexican patients with AD. Fourteen polymorphisms were genotyped in 96 subjects with AD and 100 controls; the differences in allele, genotype and haplotype frequencies were analyzed. Additionally, an ancestry analysis was conducted to exclude differences in genetic ancestry among groups as a confounding factor in the study. Significant differences in frequencies between AD and controls were found for the single-nucleotide polymorphism (SNP) rs20417 within the *PTGS2* gene. Ancestry analysis revealed no significant differences in the ancestry of the compared groups, and the association was significant even after adjustment for ancestry and correction for multiple testing, which strengthens the validity of the results. We conclude that this polymorphism plays an important role in the development of the AD pathology and further studies are required, including their proteins.

## Introduction

Alzheimer’s disease (AD) is a neurodegenerative disease which major symptom is the impairment of memory and other cognitive functions (Collette et al., 1999; Di Paola et al., 2007). It has been estimated that the global prevalence of dementia is around 4.5% and that AD accounts for 65% of the total number of dementia Alzheimer’s type (DAT; Kalaria et al., 2008; Rizzi et al., 2014), which places AD as the major global cause of dementia. The prevalence of dementia varies considerably around the world. There are few studies particularly in Mexico, however it has been determined that in Mexican population above 20 years old the prevalence of dementia is 6.1–7.9%, and the country was reported to be the fifth with the highest prevalence of the disease in Latin America (Llibre Rodriguez et al., 2008; Prince et al., 2008; Mejia-Arango and Gutierrez, 2011; Ramírez-Díaz et al., 2015). AD is a complex disease which etiology relies both in environmental and genetic factors, but its exact causes are unknown to date. The definitive diagnose of AD can be only performed by *post mortem* histological analysis, in which certain distinctive lesions must be found. These lesions consist of protein aggregates known as neuritic plaques (NPs), composed of the amyloid-β peptide (Wong et al., 1985), and neurofibrillary tangles (NFTs), composed of hyperphosphorylated tau protein (Kosik et al., 1986).

In AD the neuroinflammation is an early and continuous feature of the disease (Hensley, 2010; McGeer and McGeer, 2010; Zhang and Jiang, 2015). It has been reported that the activation of the immune system, which leads to a general inflammatory state in the brains, one of the major and most constant characteristics of AD, as well as other neurodegenerative diseases (Meraz-Ríos et al., 2013). This response involves cellular and molecular changes, the recruitment of peripheral immune cells (Rezai-Zadeh et al., 2011), and the release of inflammatory mediators in the brain (Heneka et al., 2010).

Several studies in animal models have confirmed that the presence of Aβ in the brain leads to the activation of microglial cells and astrocytes (Frautschy et al., 1992; Hanzel et al., 2014). The high levels of chemokines and chemokine receptors in brain regions surrounding NPs suggest that there is a chemotactic migration of microglia towards Aβ peptides (Walker et al., 2006). The activation of astrocytes and microglia leads to an increased secretion of pro-inflammatory proteins, such as cyclooxygenases, complement proteins and their receptors, acute phase proteins, adhesion molecules, chemokines, and cytokines (Liao et al., 2004; Ramesh et al., 2013). The chronic increased secretion of this proteins leads to increased oxidative stress and enhances cell death, which leads to neurodegeneration in the central nervous system (CNS; Meraz-Ríos et al., 2013).

It has been proposed that sequence variations in the genes that code pro-inflammatory and anti-inflammatory proteins might play a role changing the function or expression rate of the proteins and in this way modifying the inflammatory response in the brain; this could have an effect in the risk of developing AD. We selected 14 single-nucleotide polymorphisms (SNPs), according to the Alzgene Top results. Numerous studies have examined the presence of SNPs in genes of proinflammatoy cytokines such as Interleukin-1α (IL-1α; Combarros et al., 2002), Interleukin 6 (IL-6; Chen et al., 2012), Tumor Necrosis Factor α (TNF-α; Laws et al., 2005; Ardebili et al., 2011) and in the anti-inflammatory Interleukin 10 (IL-10; Bagnoli et al., 2007), whose production have found altered in CSF and peripheral blood in AD patients (Blum-Degen et al., 1995; Swardfager et al., 2010). The promoter region SNPs of TNF gene, rs1800629 and rs1799724, have been studied for a possible involvement with a functional alteration in the production of this proinflammatory cytokine, in particular the presence of the rs1799724 in Caucasians with a diagnosis of probable AD, correlated with altered levels of Aβ42 in CSF (Laws et al., 2005). IL1A is another candidate gene associated with AD (Combarros et al., 2002); in this case we selected the rs17561 variant, which has been previously studied in Americans and Japanese populations (Minster et al., 2000; Yucesoy et al., 2006). As for the anti-inflammatory cytokines, the most studied polymorphisms are rs1800871, rs1800896, rs1800872; they are located in the promoter region of IL10 gene and have been related with an alteration of transcriptional activation with a gene-dosage related effect and have replicated an association in Caucasian populations (Bagnoli et al., 2007; Combarros et al., 2009). Also, a possible interaction within these SNPs and the variants on the IL6 gene was reported (Combarros et al., 2009).

In the same way, some polymorphisms on Clusterin (CLU) and Complement Receptor 1 (CR1) genes have shown a consistent association with AD in genome-wide association studies (GWAS; Harold et al., 2009; Lambert et al., 2009; Jun et al., 2010; Hu et al., 2011). Another protein that has been linked with the inflammatory process in AD is the c-reactive protein (CRP). This acute phase reactant has been found in association with plaques and NFTs (Yasojima et al., 2000). Also, elevated tissue levels of CRP have been related with an increased risk for developing AD; however when the disease has established, the CRP levels decrease (Schmidt et al., 2002; O’Bryant et al., 2013). For these reasons, this protein has been proposed as an early biomarker for AD. Moreover, SNPs on CRP gene like rs1130864 and rs1800947 have been involved with altered CRP levels (Kok et al., 2011) and with a possible association with AD in Caucasians (Flex et al., 2004; van Oijen et al., 2007). Additionally, the cyclooxygenase-2 (COX-2) is encoded by the prostaglandin-endoperoxide synthase 2 gene (PTGS2); this enzyme involved in neuroinflammation, is responsible for prostaglandins (PG’s) synthesis. It has been documented that COX-2 expression increases in the hippocampus of AD brain, which also correlates to the severity of the pathology (Ho et al., 2001; Meraz-Ríos et al., 2013). Due to this fact, polymorphisms in the PSTG2 gene have been studied; mainly, the rs20417 located in the promoter region has shown an association with decreased risk of AD (Abdullah et al., 2006).

Recently, publications related to the presence of polymorphisms associated with the development of AD have increased; however, numerous lines of evidence have demonstrated discrepant results among populations. These findings suggest that it is necessary to reduce the confounding factors and focus on identifying the cause (Jiang et al., 2013). The vast majority of these studies have not evaluated the role of individual differences in ancestry as a confounding factor in their study population. Mexican and other Latin American populations are the result of mixed breeding among three ancestral populations: Amerindian, Caucasian and African. Intra or inter-group differences in genetic ancestry might represent a source of spurious associations in case-control studies if they are not appropriately regarded.

Considering all of the above mentioned, the aim of this study was to evaluate a set of candidate polymorphisms in inflammation-related genes, and to determine whether they are associated with the risk of developing AD in the Mexican population, considering the genetic ancestry of the study subjects.

## Materials and Methods

### Study Population

The subjects were patients with a clinical diagnose of dementia of the Alzheimer-type (DAT), or cognitively healthy subjects (controls). All subjects were Mexican, with Mexican ancestry back to the third generation, and at least 60 years old at the sampling date. This work was carried out according with the ethical standards of the Committee on Human Experimentation of the institution (Instituto Nacional de Neurología y Neurocirugía Number 100/07) in accord with the Helsinki Declaration of 1975. A Student’s *t* test was done comparing the parameters of mean age between cases and controls.

Ninety four DAT patients, previously diagnosed by a group of Geriatricians and Neurologists, were included according to the NINCDS-ADRDA (Mckhann et al., 1984) criteria. The DAT patients were interviewed at their scheduled visits at Geriatric Clinic of the General Hospital Mocel in Mexico City and Geriatric Center in Querétaro.

The inclusion criteria were: (1) patients with DAT were required to be able to read and write; (2) patients have to be 60 years old and over; and (3) they had to hand in an informed consent sheet signed by both the informant and the elderly person. The exclusion criteria were as follows: (1) acute and/or exacerbated chronic disease present within 30 days before the interview that could affect the quality of response to questionnaires, according to the medical staff of the study; (2) decreased alertness (for any cause); (3) severe aphasia; (4) visual and hearing impairment making it difficult for the patient/caregiver to fill out the questionnaires; (5) suffering other neurological diseases that could have influenced the diagnosis of dementia; and (6) live in a nursing home.

The inclusion criteria for controls were: (1) being an adult of 60 years of age or over; (2) not having memory complaint reported by either the informant or the elderly person; (3) completing an Mini Mental State Examination (MMSE) score of ≥24; (4) having the ability to read and write; and (5) handing in an informed consent sheet signed by both the informant and the older person. The exclusion criteria for this group were: (1) suffering from any acute or severe chronic illness; and (2) being less alert or suffering from severe aphasia, impaired vision and/or hearing, which would make it difficult for the older person to answer any of the questionnaires.

### Analyzed Polymorphisms

The selection of the polymorphisms was performed searching in the Alzforum database. The search was directed in order to find SNPs, located in genes that code inflammation-related proteins, and which had previous reports of association with AD in other populations.

Fourteen SNPs were selected, which were distributed among eight genes that code inflammation-related proteins. The selected genes were: *PTGS2* (Ma et al., 2008), CLU (Lambert et al., 2009), CR1 (Zhang et al., 2010), *CRP* (Eriksson et al., 2011), *TNF* (Ardebili et al., 2011), the pro-inflammatory interleukins 1α (*IL-1α*, Combarros et al., 2002) and 6 (*IL-6*, Chen et al., 2012), and the anti-inflammatory interleukin 10 (*IL-10*, Bagnoli et al., 2007). The selected SNPs are listed in Table 1.

Additionally, in order to perform the ancestry analysis of the study population, a set of 10 ancestry informative markers (AIMs) were selected (rs4884, rs2695, rs17203, rs2862, rs3340, rs722098, rs223830, rs1800498 and rs2814778). These AIMs are SNPs which frequencies significantly vary between Amerindian, Caucasian and African populations. These AIMs have already been tested to accurately estimate ancestry in populations of Latin American origin (Salari et al., 2005; Choudhry et al., 2006; Ziv et al., 2006).

**Table 1:** **SNPs analyzed in the present study**.

SNP	Chromosome	Chromosome position	Gene	SNP location	Alleles
**rs20417**	1	186681189	PTGS2	−765 Promoter	C/G
**rs9331888**	8	27611345	CLU	Exon	C/G
**rs6656401**	1	207518704	CR1	Intron	A/G
**rs1799724**	6	31574705	TNF	−850 Promoter	G/A
**rs1800629**	6	31575254	TNF	−308 Promoter	T/C
**rs1800947**	1	159713648	CRP	Exon	C/G
**rs1130864**	1	159713301	CRP	3′ UTR	G/A
**rs17561**	2	112779646	IL-1	Exon	G/T
**rs1800795**	7	22727026	IL-6	−174 Promoter	C/G
**rs1800796**	7	22726627	IL-6	−572 Pomoter	G/C
**rs1524107**	7	22728600	IL-6	Intron	G/A
**rs1800871**	1	206773289	IL-10	−819 Promoter	G/A
**rs1800896**	1	206773552	IL-10	−1082 Promoter	C/T
**rs1800872**	1	206773062	IL-10	−592 Promoter	C/A

### DNA Extraction and Genotyping

Peripheral blood was extracted from all subjects, and was stored in Vacutainer® tubes with EDTA. Genomic DNA was extracted from blood using the extraction kit Midi Kit QIAamp® DNA Blood (Qiagen). The extracted DNA was used to perform real-time PCR for the 14 inflammation related SNPs and the 10 AIMs. PCR was performed in a 7500 Fast Real Time PCR System® (Applied Biosystems). SNPs were genotyped using TaqMan® probes (Applied Biosystems) with real-time PCR detection.

### Statistical Analysis

#### Ancestry Analysis

The genotypes obtained by the genotyping of the AIMs were analyzed in order to produce estimates of the proportion in which each ancestral population is represented in the study population. To achieve this, LEADMIX (Wang, 2003) and STRUCTURE (Pritchard et al., 2000) software were required.

For the analysis with LEADMIX, the 10 genotyped AIMs were used, while the information of the frequency of these AIMs in ancestral populations was obtained from the database dbSNPs, available in the National Institute for Health website. Fisher’s exact test was used to compare proportions between cases and controls.

For the analysis with STRUCTURE, only seven of the genotyped AIMs were used (rs4884, rs2695, rs17203, rs2862, rs3340, rs1800498 and rs2814778). Because the analysis with STRUCTURE requires individual genotype information from subjects belonging to the ancestral populations (reference subjects), a database composed of data collected by Salari et al. (2005), data from the 1000 Genomes Project (Abecasis et al., 2010), and data from the study population was used. Reference subjects for the Caucasian population were Utah residents of European ancestry, Finnish, English, Spanish, Italians and Germans. Reference subjects from the African population were people from Nigeria, Sierra Leone and the Central African Republic. Because the 1000 Genomes Project lacks information from the Amerindian population, reference subjects for this population were taken exclusively from Salari et al. (2005) and they were Mayan and Native Americans from the Pima, Cheyenne and Pueblo ethnic groups.

#### Association Testing

Allele and genotype frequencies were determined using the SNPStats software (Solé et al., 2006). Departure from Hardy–Weinberg equilibrium (HWE) was assessed by *χ*^2^ test, using the GenAlEx 6.5 software (Peakall and Smouse, 2012).

For the association testing, estimates of the individual ancestry proportions obtained from STRUCTURE were included along with age and gender in the regression model for odds ratio calculation in SNPStats. For this analysis, only the values for Amerindian and African ancestry were included to avoid collinearity in the model. In order to account for multiple testing, the Bonferroni correction was applied while interpreting the significance of the associations found.

#### Linkage Disequilibrium Analysis

Additionally the level of linkage disequilibrium (LD) among the selected SNPs was calculated. For this purpose, the Haploview v. 4.2 software was used.

## Results

### Age and Gender in the Study Population

A total of 194 subjects were genotyped, 94 DAT and 100 controls. Table 2 presents the distribution of gender among each study group as well as the mean age for each gender and group. No significant differences were observed in the distribution of gender between study groups (*p* = 0.543). Results from the Student’s *t* test comparing the mean age between DAT and controls not showed significant differences (*p* = 0.111). Also, we didn’t find differences when we performed a comparison testing the mean age of the different genders independently (women *p* = 0.071, men *p* = 0.899).

**Table 2:** **Age and gender in the study population**.

Study group	Number of women	Mean age women (yrs)	Number of men	Mean age men (yrs)	Total mean age
**DAT**	65 (69%)	76.37 ± 1.11	29 (31%)	74.93 ± 1.68	75.93 ± 0.92
**Controls**	64 (64%)	73.46 ± 1.15	36 (36%)	74.67 ± 1.29	73.9 ± 0.86

### Ancestry Analysis of the Study Population

#### Ancestry Proportion Estimation in Study Groups

**Figure 1:**
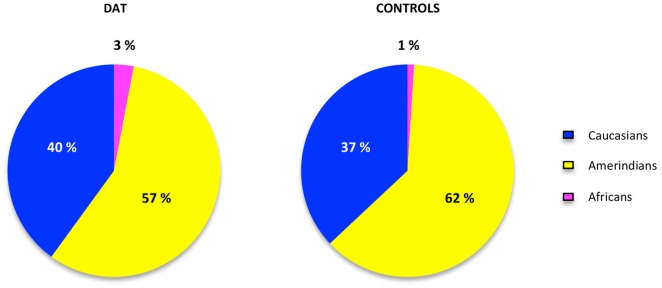
**Ancestry proportions in cases and controls.** The proportions of ancestry in each group of study were estimated in the LEADMIX software (Wang, 2003) taking the ancestral populations information of 10 AIMs obtained from the database of the National Institute for Health website.^1^ No significant differences were found after the Fisher’s exact test (*p* = 0.706).

**Figure 2:**
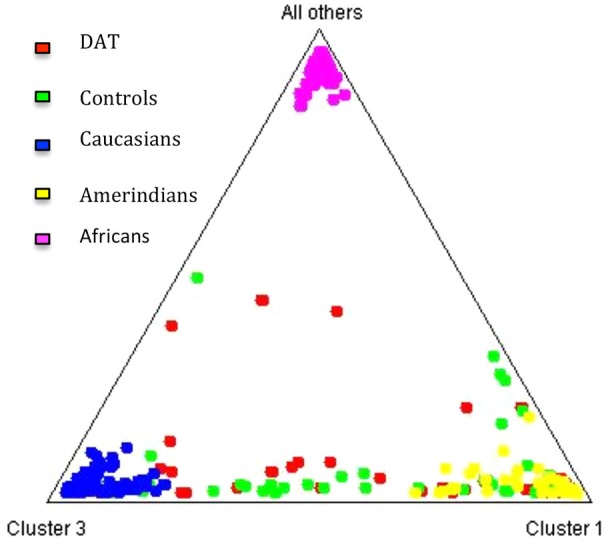
**Cluster analysis of the three ancestral populations and the study groups**. The triangle obtained from the STRUCTURE software (Pritchard et al., 2000) allows to observe that the distribution of the individuals of each study group between the three ancestral clusters is similar.

The contribution of each ancestral population in each study group was calculated using the Bertorelle and Excoffier (1998) estimator with LEADMIX. Results are shown in Figure 1. As it can be noted, Amerindian ancestry was represented in a higher proportion in controls as well as in DAT, followed closely by Caucasian ancestry, and finally by African ancestry in a lower proportion. The results of the Fisher’s exact test showed that there are no significant differences (*p* = 0.706) in ancestry proportions between DAT and controls. This result was corroborated with the analysis in STRUCTURE, which yielded the ancestry proportion estimates for each study subject; the estimates were subsequently grouped into a cluster (Figure 2). Derived from these analyses we conclude that both groups are genetically homogeneous in ancestry.

### Analyzed SNPs and AD Risk

Allele and genotype frequencies of the 14 SNPs were analyzed (Table 3). Genotypes were assessed for departure from HWE. Four polymorphisms presented a significant departure in the control group; they were rs6656401 (*p* = 0.012), rs1799724 (*p* = 0.013), rs1800795 (*p* = 0.043) and rs1800871 (*p* = 0.000). Because of the deviations, those SNPs were excluded from our replication study.

**Table 3:** **Allelic and genotyping analysis**.

SNP	Allele frequencies		Genotype frequencies			Association with response status (adjusted by sex + age)			*p* (adjusted by sex + age + ancestry)
						*p*	OR	95% CI
**rs20417**	**C**	**G**	**CC**	**CG**	**GG**				
AD	0.08	0.92	0.01	0.14	0.85	0.0018**	2.99	1.47–6.11	<0.0001**
Control	0.19	0.81	0.03	0.32	0.65
**rs9331888**	**G**	**C**	**GG**	**GC**	**CC**
AD	0.46	0.54	0.17	0.59	0.24	0.99	1.00	0.52–1.93	0.48
Control	0.53	0.47	0.31	0.44	0.25
**rs6656401**	**A**	**G**	**AA**	**AG**	**GG**
AD	0.16	0.84	0.05	0.21	0.73	+	+	+	+
Control	0.2	0.8	0.08	0.24	0.68
**rs1799724**	**A**	**G**	**AA**	**AG**	**GG**
AD	0.2	0.8	0.05	0.29	0.66	+	+	+	+
Control	0.32	0.68	0.16	0.33	0.51
**rs1800629**	**T**	**C**	**TT**	**TC**	**CC**
AD	0.1	0.9	−	0.19	0.81	0.16	0.56	0.25–1.28	0.7
Control	0.06	0.94	-	0.11	0.89
**rs1800947**	**G**	**C**	**GG**	**GC**	**CC**
AD	0.04	0.96	0.01	0.06	0.93	0.76	0.84	0.27–2.62	0.07
Control	0.03	0.97	-	0.06	0.94
**rs1130864**	**A**	**G**	**AA**	**AG**	**GG**
AD	0.35	0.65	0.13	0.45	0.43	0.27	1.39	0.77–2.51	0.0008**
Control	0.4	0.6	0.15	0.51	0.34
**rs17561**	**T**	**G**	**TT**	**TG**	**GG**
AD	0.26	0.74	0.06	0.38	0.55	0.31	1.34	0.76–2.38	0.52
Control	0.3	0.7	0.97	0.45	0.48	
**rs1800795**	**G**	**C**	**GG**	**GC**	**CC**
AD	0.18	0.82	0.05	0.24	0.7	+	+	+	+
Control	0.1	0.9	0.03	0.15	0.82
**rs1800796**	**C**	**G**	**CC**	**CG**	**GG**
AD	0.32	0.68	0.09	0.47	0.45	0.69	1.13	0.63–2.01	0.27
Control	0.36	0.64	0.16	0.41	0.43
**rs1524107**	**A**	**G**	**AA**	**AG**	**GG**
AD	0.31	0.69	0.09	0.46	0.46	0.88	1.04	0.59–1.85	0.02
Control	0.34	0.66	0.15	0.39	0.46
**rs1800871**	**A**	**G**	**AA**	**AG**	**GG**
AD	0.44	0.56	0.21	0.46	0.33	0.87	1.05	0.57–1.93	0.87
Control	0.42	0.57	0.17	0.51	0.32
**rs1800896**	**T**	**C**	**TT**	**TC**	**CC**
AD	0.46	0.54	-	0.91	0.09	+	+	+	+
Control	0.46	0.54	-	0.91	0.09
**rs1800872**	**A**	**C**	**AA**	**AC**	**CC**
AD	0.39	0.61	0.16	0.46	0.38	0.4	1.29	0.71–2.34	0.33
Control	0.42	0.58	0.16	0.52	0.32

** After Bonferroni correction (p = 0.00357) only the rs20417, was significant. + Excluded because of significant departure from Hardy-Weinberg in controls.

In order to adjust for multiple testing, the Bonferroni correction was applied; according to the calculations, a corrected *p* value less than 0.00357 was considered significant. The association test showed important differences in the rs20417 located in the *PTGS2* gene. The presence of GG genotype was significantly more frequent in the DAT group (OR = 2.89, 95% CI = 1.41–5.92, *p* = 0.0028), this could suggest an association with AD. No significant differences were found between DAT and controls in the allele frequencies of the rest of the SNPs analyzed (Table 3).

#### LD Calculation

The software Haploview was used to measure LD among pairs of *loci*, and the *r*^2^ an LD parameter was calculated.

**Figure 3:**
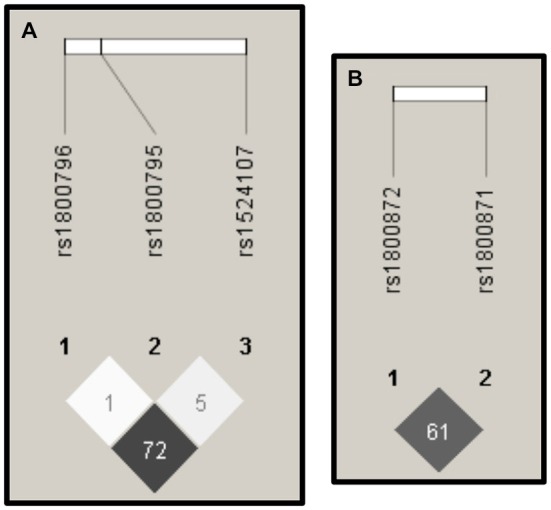
**Linkage Disequilibrium (LD).** The images derived from the Haploview software (Barrett et al., 2005) display the *r*^2^ values of LD. High values of *r*^2^ (depicted in dark gray) were presented among SNPs in IL-6 **(A)** and IL-10 **(B)**.

Figure 3 shows that the highest values of *r*^2^ can be found between the SNPs rs1800796 and rs1524107 within the *IL-6* gene, and between the SNPs rs1800871 and rs1800872 within the *IL-10* gene.

## Discussion

This study is focused on the search of genetic risk factors associated with AD in Mexican population, considering the ancestry of the population studied and analyzing some of the main genes related to the inflammatory process observed in AD patients. Previous studies have identified AD associated variants in the genes analyzed in this study; nevertheless, until now these studies have been often limited to the evaluation of variants within a single gene, or a few genes.

HWE has been proposed as a routine evaluation in case-control genetic association studies. Genotyping errors, population stratification, a small sample size and non-random mating generally contribute to departures on this equilibrium and favor false associations (Salanti et al., 2005; Wang and Shete, 2012). Based in this fact, we omit four SNPs from the association test, because they present a HWE departure in control group. Another resource that we used for reducing the probability of obtaining false positives in the statistical analysis of the association test was the correction for multiple testing.

In this study, the homozygous genotype GG in the SNP rs20417, was overrepresented in AD patients and underrepresented in controls in the *PTGS2* gene. These results essentially match with the results obtained by Abdullah et al. (2006) and Listì et al. (2010), who found the C allele is linked to a decrease in the risk of developing the disease. Our data suggest that GG polymorphism in *PTGS2* could be a risk factor for AD in Mexican population.

*PTGS2* is expressed in neurons of the neocortex and hippocampus under normal physiological conditions, suggesting its essential role in normal neuronal function. COX-2 catalyzes the formation of PG’s, which are well-known inflammatory mediators. In fact, during some pathological conditions it can be expressed in microglia and astrocytes. Several authors have noted that COX2 is involved in several neurological diseases such as Parkinson’s disease (Teismann et al., 2003), amyotrophic lateral sclerosis (Almer et al., 2001), schizophrenia (Müller et al., 2004) and AD (Pasinetti and Aisen, 1998; Hoozemans and O’Banion, 2005). In 1999, Yasojima et al. reported that COX-2 was substantially upregulated in affected areas of AD brain, this dominant upregulation in neurons could reflect a defensive response of the neurons to stress (Yasojima et al., 1999). However, the chronic expression of COX-2 may contribute to aggravate stress through the generation of free radicals causing oxidative stress and neurotoxicity. COX-2 in the CNS may enhance neuritic plaque formation by means of the production of PGE2, which can induce the expression of the Aβ precursor protein (APP). In turn, the presence of NPs can increase the expression of COX-2 (Nogawa et al., 2003).

Therefore, the GG genotype in the SNP rs20417 that displayed association with AD in this study might have an effect over the gene expression rate, resulting in an alteration of protein concentrations. This might exert an influence over the mechanisms of Aβ synthesis, thus contributing to modify the risk of developing the disease. Conversely, the C allele has a reduced promoter activity, suggesting that lower promoter activity of COX-2 gene might be protective for AD. In the rest of the analyzed SNPs no significant differences were found when comparing the allele or genotype frequencies between AD patients and controls. These results contradict previous reports in which an association between these SNPs and the risk of developing AD was found (Bonafè et al., 2001; Brull et al., 2001; Combarros et al., 2002, 2009; Flex et al., 2004; Laws et al., 2005; Bagnoli et al., 2007; van Oijen et al., 2007; Harold et al., 2009; Lambert et al., 2009; Jun et al., 2010; Zhang et al., 2010; Hu et al., 2011; Chen et al., 2012; Shen et al., 2014).

We also found two regions of high LD, one in the *IL-6* gene and another in the *IL-10* gene. These results and the HWE deviations presented in both genes, suggest that future genetic association studies focused on those regions should be done using a larger sample size in order to clarify a possible association with AD in the Mexican population as was observed principally in Caucasian populations (Bonafè et al., 2001; Brull et al., 2001; Bagnoli et al., 2007; Combarros et al., 2009; Chen et al., 2012).

Most inconsistencies in genetic association studies, are probably due to ethnic differences among analyzed populations (Dixon et al., 2011), because populations with differences in genetic ancestry should not be extrapolated. In order to validate our results, we focused in the stratification of our samples, which is a confounding factor that could partly explain the relative lack of replication across populations (Thomas and Witte, 2002). To avoid the effects of stratification we estimate the ancestry proportion in each study group and all the groups were included with age and gender in the regression model for odds ratio calculation in SNPStats. The inclusion of the adjustment for ancestry in the association testing ensures that the contribution of each ancestral population is homogeneous between AD patients and controls and it does not play a confounding role in the analysis (Epstein et al., 2007). The ancestry proportion estimates obtained in this study are comparable with previous reports in the Mexican population, observed by Juárez-Cedillo et al. (2008) using 15 short tandem repeats (STRs), and by Rangel-Villalobos et al. (2009), using 12 Y-chromosome STRs. Therefore these results corroborate the validity of the AIMs selected.

It is important to mention that this is the first study in Mexican population that considers the analysis of ancestry in AD patients in which it was possible to find that the presence of the GG genotype in the SNP rs20417 displayed an association with AD patients in our population. We consider the association of rs20417 genotype GG was adequate based on confidence interval (1.47–6.11). In the same way after the correction by ancestry, another polymorphism (rs1130864) showed a significant *p* value (0.0008), however this association is questionable because the IC = 0.77–2.51. Although this could be a pilot study, it is essential to increase the sample size to support statistical power. Also, it is important to conduct more extensive studies involving genes related to the COX2 metabolism. This will establish possible mechanisms involved in the pathological process of the disease.

## Conclusions

In summary, this is the first study in Mexican population that considers the analysis of ancestry in AD patients. Our results showed that the rs20417 SNP in the *PTGS2* gene presented differences in its allele and genotype frequencies pointing at the G allele and the GG genotype, and it could be considered as a risk factor for AD in Mexican patients. These results manifest the relevance of the role that COX-2 is playing in the pathological mechanisms that result in the development of AD. Ancestry analysis showed that there were not significant differences in AD patients and controls with respect to genetic ancestry, corroborating the validity of the associations found.

## Conflict of Interest Statement

The authors declare that the research was conducted in the absence of any commercial or financial relationships that could be construed as a potential conflict of interest.

## Footnotes

^1^http://www.ncbi.nlm.nih.gov/SNP
